# Mutations in the Arabidopsis *ROL17*/*isopropylmalate synthase 1* locus alter amino acid content, modify the TOR network, and suppress the root hair cell development mutant *lrx1*

**DOI:** 10.1093/jxb/ery463

**Published:** 2019-02-08

**Authors:** Myriam Schaufelberger, Florian Galbier, Aline Herger, Rita de Brito Francisco, Stefan Roffler, Gilles Clement, Anouck Diet, Stefan Hörtensteiner, Thomas Wicker, Christoph Ringli

**Affiliations:** 1 Institute of Plant and Microbial Biology, Zurich-Basel Plant Science Center, University of Zurich, Zurich, Switzerland; 2 Institute of Molecular Plant Biology, Zurich-Basel Plant Science Center, Zurich, Switzerland; 3 Institute of Plant Sciences Paris-Saclay, CNRS, Université Paris Diderot, INRA, Université Paris Sud, Université d’Evry, Université Paris-Saclay, Rue de Noetzlin, Gif-sur-Yvette, France; 4 Institut Jean-Pierre Bourgin, INRA, AgroParisTech, CNRS, Université Paris-Saclay, Versailles, France

**Keywords:** Arabidopsis, AZD-8055, IPMS1, leucine, extensin, LRX1, *rol17*, root, root hair, TOR

## Abstract

The growth and development of organisms must be tightly controlled and adjusted to nutrient availability and metabolic activities. The Target of Rapamycin (TOR) network is a major control mechanism in eukaryotes and influences processes such as translation, mitochondrial activity, production of reactive oxygen species, and the cytoskeleton. In *Arabidopsis thaliana*, inhibition of the TOR kinase causes changes in cell wall architecture and suppression of phenotypic defects of the cell wall formation mutant *lrx1* (*leucine-rich repeat extensin 1*). The *rol17* (*repressor of lrx1 17*) mutant was identified as a new suppressor of *lrx1* that induces also a short root phenotype. The *ROL17* locus encodes isopropylmalate synthase 1, a protein involved in leucine biosynthesis. Dependent on growth conditions, mutations in *ROL17* do not necessarily alter the level of leucine, but always cause development of the *rol17* mutant phenotypes, suggesting that the mutation does not only influence leucine biosynthesis. Changes in the metabolome of *rol17* mutants are also found in plants with inhibited TOR kinase activity. Furthermore, *rol17* mutants show reduced sensitivity to the TOR kinase inhibitor AZD-8055, indicating a modified TOR network. Together, these data suggest that suppression of *lrx1* by *rol17* is the result of an alteration of the TOR network.

## Teaser

Interfering with leucine biosynthesis affects the TOR network and causes changes in cell growth that lead to suppression of the cell wall formation mutant *lrx1*.

## Introduction

Owing to their sessile nature, plants have a particularly strong need to monitor environmental conditions to optimize growth and development. This optimization is not limited to the targeted uptake of nutrients but includes adjusting metabolic activities to the available resources. The Target of Rapamycin (TOR) network controls eukaryotic cell growth by sensing nutrient availability and growth factors, and integrates these signals to adjust a wide range of cellular processes such as transcriptional and translational activities, autophagy, mitochondrial activity, and the dynamics of the cytoskeleton (Leith and Hall, 2011; Dobrenel *et al.*, 2016*a*). The phosphatidyl inositol 3-kinase-like serine/threonine kinase TOR is central to the TOR network; *tor* knockout mutations are lethal in many species, including *Arabidopsis thaliana*, underlining the importance of this protein (Menand *et al.*, 2002; Laplante and Sabatini, 2012). Rapamycin, a TOR kinase-specific inhibitor, is instrumental in the characterization of the TOR network and is used in medical applications owing to its growth-inhibiting, anti-tumor, and immunosuppressive properties (Huang *et al.*, 2003; Martelli *et al.*, 2018). Rapamycin inhibits the TOR kinase by forming a ternary complex with FKBP12 (FK506 binding protein 12) and TOR. Owing to amino acid polymorphisms, however, the FKBP12 of Arabidopsis and other plants is not very effectively recruited into this complex; expression of, for example, the human or yeast *FKBP12* renders Arabidopsis more rapamycin sensitive (Mahfouz *et al.*, 2006; Sormani *et al.*, 2007). The development of a new generation of FKBP12-independent, TOR-specific ATP-competitive active-site inhibitors such as AZD-8055 circumvents this problem and provides excellent tools for more direct analysis of the TOR network in plants by pharmaceutical means (Chresta *et al.*, 2010; Liu *et al.*, 2011; Montané and Menand, 2013; Dobrenel *et al.*, 2016*b*; Schepetilnikov *et al.*, 2017). As an alternative strategy, RNA-mediated silencing of the *TOR* gene has been used to interfere with TOR signaling (Deprost *et al.*, 2007; Caldana *et al.*, 2013) and revealed genes and metabolites that are under the influence of the TOR network. The identification of mutations in genes encoding TOR-interacting proteins such as LST8 (lethal with Sec13-8) or RAPTOR have been helpful in characterizing the TOR network (Anderson *et al.*, 2005; Deprost *et al.*, 2005; Ahn *et al.*, 2011; Moreau *et al.*, 2012). Modulation of the activity of proteins involved in the TOR network frequently leads to an alteration in the sensitivity of the TOR network to TOR kinase inhibitors. This characteristic feature has been used in chemical genetic screens to identify components or processes that are functionally connected to the TOR network. The genes identified in this way have diverse functions including transcriptional regulation, amino acid metabolism, and tRNA maturation (Chan *et al.*, 2000; Goehring *et al.*, 2003; Li *et al.*, 2015).

Metabolomic and transcriptomic analyses have revealed that interfering with the TOR kinase has a broad impact on primary (e.g. the tricarboxylic acid cycle) and secondary (e.g. flavonoids) metabolism (Moreau *et al.*, 2012; Ren *et al.*, 2012; Caldana *et al.*, 2013). Its inhibition interferes with cell growth and induces autophagy, a process by which cellular components are degraded to recycle nutrients (Liu and Bassham, 2010). Together with a concomitant inhibition of translation, this causes an increase in the amino acid content of the cell. The energy-intensive translational machinery is a major target of the TOR pathway (Laplante and Sabatini, 2012; Dobrenel *et al.*, 2016*b*), and mutual influence of the TOR network and amino acid levels has been demonstrated (Dobrenel *et al.*, 2016*a*).

Plant cell growth is driven by turgor pressure exerted by the cell and limited by the expansion of the cell wall that surrounds each cell (Cosgrove, 2014). The expression of cell-wall-related genes and the cell wall architecture are modified upon altering the activity of the TOR network by genetic or pharmaceutical means (Leiber *et al.*, 2010; Ren *et al.*, 2012; Caldana *et al.*, 2013). Leucine-rich repeat extensins (LRXs) are extracellular proteins involved in cell wall formation, and mutations in the *LRX* genes cause changes in cell wall composition and ultrastructure (Draeger *et al.*, 2015; Fabrice *et al.*, 2018). Analysis of LRX proteins expressed in different tissues revealed that they act as extracellular receptors of RALF (rapid alkalinization factor) peptides (Mecchia *et al.*, 2017), and function together with the *Catharanthus roseus*-like receptor kinase FERONIA (Haruta *et al.*, 2014; Dünser *et al.*, 2018) to establish a link between the cell wall and the cytoplasm. Suppression of the Arabidopsis *lrx1* mutant phenotype by interfering with TOR signaling suggests that the LRX-related process is under the influence of the TOR network (Leiber *et al.*, 2010).

The observed suppression of *lrx1* by alteration of the TOR network led us to investigate whether new TOR signaling components can be identified using suppression of *lrx1* and altered sensitivity to the TOR kinase inhibitor AZD-8055 as parameters for selection. Here, we describe the characterization of *rol17*, which suppresses *lrx1* and shows reduced sensitivity to AZD-8055. The *rol17* locus encodes isopropyl malate synthase 1 (IPMS1), an enzyme involved in leucine (Leu) biosynthesis. Metabolomic analysis revealed that the effect of *rol17* does not correlate with reduced Leu accumulation, suggesting that IPMS1 might be involved in establishing a link between amino acid biosynthesis and the TOR network that is required to achieve coordinated plant growth and development.

## Materials and methods

### Plant growth and molecular markers

*Arabidopsis thaliana*, ecotype Columbia (Col), was used for all experiments. The SAIL line *rol17-2* is in the *qrt1-2* mutant background (Sessions *et al.*, 2002), which required the *qrt1-2* mutant to be used as the wild-type control of *rol17-2*. Seeds were sterilized for 10 minutes with 1% sodium chlorite, 0.03% Triton X-100, washed three times with sterile water, and then grown on Murashige and Skoog (MS) medium [0.5 × MS, 2% sucrose, 100 μg/ml myo-inositol, 0.6% phytagel (Sigma)] or on Hoagland (HG) medium (Barberon *et al.*, 2008), in a growth chamber at 22 °C, with a 16 h/8 h light/dark cycle, in vertical orientation. For crossing and propagation, seedlings were planted in soil and grown under the same conditions. The T-DNA insertion lines were obtained from the Nottingham Arabidopsis Stock Center and were produced as described by Alonso *et al.* (2003). The ethyl methanesulfonate (EMS) mutagenesis of *lrx1* was previously described by Diet *et al.* (2006). Detection of the EMS-induced point mutations and the T-DNA alleles was done by PCR, using the primers listed in Supplementary Tables S2 and S3 at *JXB* online.

### Phenotypic analysis of seedlings

The root hair phenotype was analyzed with an MZ125 stereomicroscope (Leica) and images were obtained with a DFC420 digital camera (Leica). For root length measurements, seedlings were grown as described above, the plates were scanned, and root length was measured using ImageJ software. On one plate, two genotypes were grown on a single lane, which was always at the same position on the plates (same distance from the top), to avoid positional effects that can influence plant growth. Several plate replicates were used to produce the data points.

### AZD-8055 treatment

AZD-8055 was dissolved in DMSO and added to the MS medium (described above) after autoclaving. Sterilized seeds were directly plated, germinated, and grown on medium containing AZD-8055 for 7 days. For the control treatment without AZD-8055, only DMSO was added to the medium.

### Whole-genome sequencing

For whole-genome sequencing, 10 seedlings of an F2 population segregating for *rol17* and showing a wild type-like phenotype were isolated, and the phenotype was confirmed in the F3 generation. Fifty seedlings of each of the 10 F3 families were pooled, ground in liquid nitrogen, and DNA was extracted following an established protocol (Fulton *et al.*, 1995). In parallel, DNA of the *lrx1* mutant was also extracted. DNA sequencing corresponding to a 20-fold coverage was outsourced (BGI Tech Solutions, Hong Kong) and obtained for the *lrx1* mutant and the *lrx1 rol17* double mutant. Sequences of the *lrx1* and *lrx1 rol17* mutants were separately mapped to the Arabidopsis genome (available on TAIR, https://www.arabidopsis.org/; last accessed Jan 2019), and polymorphisms to the *lrx1* sequence were subtracted from those to the *lrx1 rol17* mutant. The resulting list of *rol17*-specific single nucleotide polymorphisms (SNPs) was filtered for mutations changing the sequence of encoded proteins based on the annotation provided by the TAIR database. For the SNP selection we used in-house Perl scripts (which can be provided upon request).

### RNA isolation and RT–PCR

Seedlings were grown for 8 days on MS medium as described above in a vertical orientation, frozen in liquid nitrogen, and ground with a mortar and pestle. Approximately 100 mg fresh material was used for RNA isolation using the total RNA isolation system (Promega). The RNA was quantified by using ND-1000 (Nanodrop) and 300 ng was used for reverse transcription using the iScript kit (Bio-Rad). The DNA produced was diluted 10-fold in nuclease-free water and 1 µl was used for PCR, using primers specific for *IPMS1* and *ACTIN2* (for primer sequences, see Supplementary Table S4).

### Targeted Leu analysis by LC-MS

For comparison of Leu levels, entire seedlings grown for 8 days in a vertical orientation on either 0.5 × MS or HG medium containing phytagel (Sigma) and Ultrapure Agarose (Invitrogen), respectively, were collected. Per sample, 100 mg of fresh material was frozen in liquid nitrogen and ground with glass beads in a Retsch mill. Polar compounds were extracted with 70% methanol and 1 µg ml^–1^ of the internal standard DL-2-aminoheptanedioic acid. The samples were briefly vortexed and centrifuged at 15 000 × *g* for 15 min. The collected supernatants were fully evaporated in a Savant SpeedVac concentrator (Thermo Fisher Scientific) at 42 °C, resuspended in 30 μl of 50% acetonitrile, and transferred to liquid chromatography (LC) vials. Leu quantification was performed using an ultra-performance LC (UPLC) system (Thermo Scientific Dionex UltiMate 3000) coupled to a Bruker Compact electrospray ionization quadrupole time-of-flight mass spectrometer (Bruker Daltonics). The UPLC separation was performed with a C18 reverse-phase column (ACQUITY UPLC ^TM^ BEH C18, 1.7 µm, 2.1 × 150 mm; Waters) at 45 °C using the following gradient of solvent A [acetonitrile, 0.1% (v/v) formic acid] and solvent B [H_2_O, 0.1% (v/v) formic acid]: 0–0.1 min, 99% A; 0.1–7 min, 30% A; 7.1–10 min, 99% A. The flow rate was 0.3 ml min^–1^ and 5 µl of each sample was injected. The electrospray ionization source was operated in positive mode and parameters were set as follows: gas temperature 220 °C, drying gas 9 l min^–1^, nebulizer 2.2 bar, capillary voltage 4500 V, and end plate offset 500 V. The instrument was set to acquire m/z 50–1300. All data were analyzed using DataAnalysis (version 4.2, Bruker Daltonics) and TargetAnalysis (version 1.3, Bruker Daltonics). Absolute Leu quantification was based on a standard curve between 0.06 and 1 µg. This analysis was performed using QuantAnalysis software (version 2.2, Bruker Daltonics).

### Metabolomics analysis by GC-MS

Seedlings were grown for 8 days in vertical orientation on HG medium supplemented with 1% glucose and 0.8% UltraPure Agarose (Invitrogen), and 100 mg of fresh tissue was collected, frozen in liquid nitrogen, and ground in a Retsch mill. A 50 mg sample of the ground powder was resuspended in 1 ml of –20 °C precooled water:acetonitrile:isopropanol (2:3:3, v:v:v) containing the internal standard ribitol at 4 µg ml^–1^ (Fiehn *et al.*, 2008) vortexed, and then shaken at 1400 rpm for 10 min at 4 °C in an Eppendorf thermomixer. After centrifugation, 100 µl was dried overnight in a Savant SpeedVac concentrator (Thermo Fisher Scientific) at 35 °C. A 10 µl volume of 20 mg ml^–1^ methoxyamine (Sigma) containing 10 µg ml^–1^ of a C8 to C30 fatty acid methyl esters (FAMES) mixture was added and the samples were shaken for 90 minutes at 1400 rpm and 28 °C in an Eppendorf thermomixer. Next, 90 µl of *N*-methyl-*N*-trimethylsilyl-trifluoroacetamide (Aldrich) was added and the reaction continued for 30 min at 37 °C. After cooling, 50 µl was transferred to an Agilent vial for injection. Four hours after derivatization, 1 µl of sample was injected in splitless mode on an Agilent 7890A gas chromatograph coupled to an Agilent 5977B mass spectrometer. The column was an Rxi-5SilMS (30 m with 10 m Integra-Guard column, Restek). The liner (Restek) was changed before each series analysis of 24 samples. The oven temperature was 70 °C for 7 min and then increased at 10 °C min^–1^ to 330 °C for 4 min (run length 36.5 min). Helium constant flow was 0.7 ml min^–1^. Temperatures were the following: injector 250 °C, transfer line 300 °C, source 230 °C, and quadrupole 150 °C. Samples and blanks were randomized. Three independently derivatized quality controls (pools of all the samples) were injected at the beginning, middle, and end of the analysis for monitoring the derivatization stability. An alkane mix (C10, C12, C15, C19, C22, C28, C32, C36) was injected in the middle of the queue for external retention index (RI) calibration and FAMES (C8 to C30) were added to each sample for internal RI calibration. Five scans per second were acquired. After the splitless analysis was performed, samples were injected in split mode (1:30) to avoid saturation of abundant compounds. Raw Agilent data files were converted to NetCDF format and analyzed with AMDIS (http://chemdata.nist.gov/mass-spc/amdis/). A home retention indices/mass spectra library built from the NIST (Agilent), Golm (Kopka *et al.*, 2005), and Fiehn (Zhou *et al.*, 2007) databases and standard compounds were used for metabolite identification. After validation of the identifications, a more accurate quantification was performed on a chosen ion trace using TargetLynx software (Waters) after conversion of the NetCDF file to MassLynx format (Databridge software, Waters). Statistical analysis was performed with TMEV. Univariate analysis by permutation (one-way and two-way ANOVA) was first used to select the significant metabolites (*P*<0.01). Multivariate analysis (hierarchical clustering and principal component analysis) was then carried out on the selected metabolites. The concentrations of 77 metabolites were expressed in µg mg^–1^ fresh weight in the following way: ribitol (internal standard) normalized peak area was calibrated to the response coefficient to ribitol of the standard of each of these 77 metabolites (one point in splitless mode and one point in split mode to ensure that the response was linear). This one-point calibration gives a good estimation of the absolute concentration.

## Results

### *rol17* suppresses *lrx1* and reduces sensitivity to the TOR inhibitor AZD-8055

**Fig. 1.**
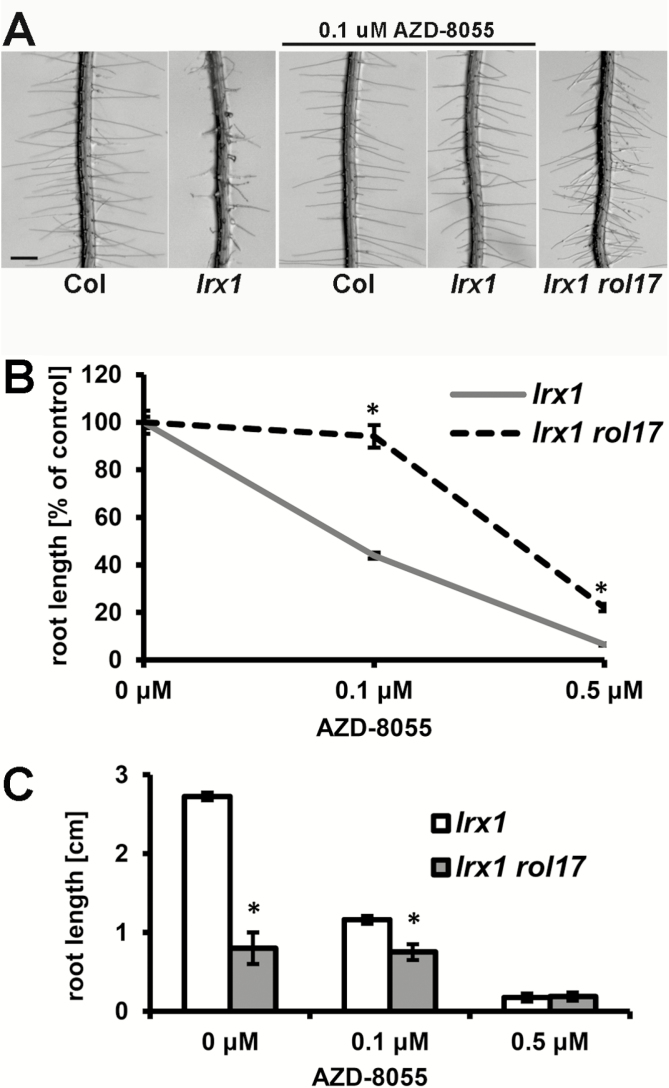
*rol17* suppresses *lrx1* and causes hyposensitivity to AZD-8055. (A) When seedlings are grown on agar plates in vertical orientation, root hairs form regularly in the wild type (Col) but are deformed and often collapsed in the *lrx1* mutant. The *lrx1 rol17* mutant develops wild type-like root hairs. Bar=0.5 mm. (B) Root length of seedlings grown for 7 days in the presence of increasing concentrations of AZD-8055. The identified *lrx1 rol17* mutant is less sensitive to AZD-8055, showing less reduction in root elongation (relative to growth without AZD-8055) compared with *lrx1*. Asterisks indicate significant differences (*P*<0.01; *t*-test, *n*>20). Error bars represent SEM. (C) *lrx1 rol17* mutants develop shorter roots than *lrx1* at concentrations of 0 and 0.1 μM AZD-8055. Seedlings were grown for 7 days in vertical orientation. Asterisks indicate significant differences (*P*<0.01; *t*-test, *n*>20). Error bars represent SEM.

The *lrx1* mutant of Arabidopsis is characterized by a defect in root hair development but shows otherwise normal root growth because *LRX1* is predominantly expressed in the root hairs (Baumberger *et al.*, 2001). Wild-type Col seedlings have regular and long root hairs, whereas the root hairs of the *lrx1* mutant are misshapen, short, and frequently burst (Fig. 1A). To obtain mutants in which the *lrx1* root hair phenotype was suppressed, *lrx1* seeds were mutagenized with EMS. In the M2 generation, *lrx1 rol* (*repressor of lrx1*) mutant seedlings that developed wild type-like root hairs—that is, a suppressed *lrx1* phenotype—were selected, and their phenotype was confirmed in the M3 generation (Diet *et al.*, 2006). As inhibiting TOR kinase with rapamycin in seedlings results in altered cell wall development and the suppression of *lrx1* (Leiber *et al.*, 2010), we wanted to test whether some of the *lrx1 rol* mutants identified were affected in the functioning of the TOR network, using sensitivity to a TOR kinase inhibitor as a parameter. The new-generation TOR kinase inhibitor AZD-8055, which causes a reduction in root elongation (Montané and Menand, 2013), was used in this experiment. As shown in Fig. 1A, AZD-8055 also suppresses the *lrx1* root hair phenotype, suggesting a comparable effect of rapamycin (as previously shown by Leiber *et al.*, 2010) and AZD-8055. To test for AZD-8055 sensitivity, root length was used as a parameter. Among the identified mutants, the *lrx1 rol17* line showed reduced sensitivity to AZD-8055. *lrx1* seedlings responded to low concentrations (0.1 μM) of AZD-8055, while *lrx1 rol17* seedlings were not affected at this concentration. Even at higher concentrations (0.5 μM), *lrx1 rol17* seedlings showed less reduction in root length than *lrx1* seedlings (Fig. 1B). This observation suggests that the *rol17* mutation causes an alteration of the TOR network, and led us to pursue the characterization of the *lrx1 rol17* line. *lrx1* single mutants are not affected in terms of growth of the main root (Baumberger *et al.*, 2003). However, even in the absence of AZD-8055, *lrx1 rol17* mutant seedlings developed shorter roots than *lrx1* (Fig. 1C), indicating that *rol17* negatively affects root growth. With regard to root hair development, the *lrx1* root hair formation defect is fully suppressed in the *lrx1 rol17* mutant (Fig. 1A).

Backcrossing of *lrx1 rol17* with the parental *lrx1* line produced F1 seedlings with an *lrx1* phenotype, indicating that the *rol17* mutation is recessive in nature. This was confirmed in the F2 generation, where the *lrx1* and wild type-like phenotypes segregated in a 3:1 ratio (206 *lrx1*-like seedlings and 54 *lrx1 rol17* seedlings).

### *ROL17* encodes isopropyl malate synthase 1

**Fig. 2.**
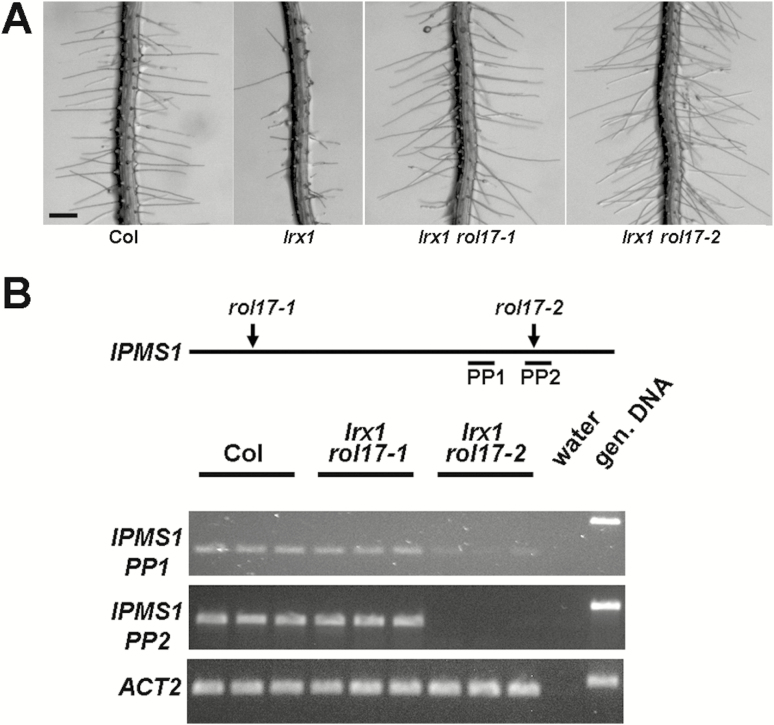
Both *rol17* alleles suppress *lrx1* but show differences in gene expression. (A) *rol17-1* and *rol17-2* result in comparable suppression of the *lrx1* root hair phenotype. Eight-day-old seedlings grown in vertical orientation are shown. Wild-type (Col) and *lrx1* roots are shown for comparison. Bar=0.5 mm. (B) Scheme of *IPMS1* showing the positions of the point mutation of *rol17-1* and the T-DNA insertion site of *rol17-2*. The primer pairs (PP) used for RT–PCR amplification are indicated, with PP2 primers flanking the T-DNA insertion site in *rol17-2*. Expression levels were tested by semi-quantitative RT–PCR on RNA extracted from 7-day-old seedlings. Amplification of the *ACTIN2* (*ACT2*) gene was used as an internal standard to confirm the use of comparable amounts of RNA as starting material in the different samples.

**Fig. 3.**
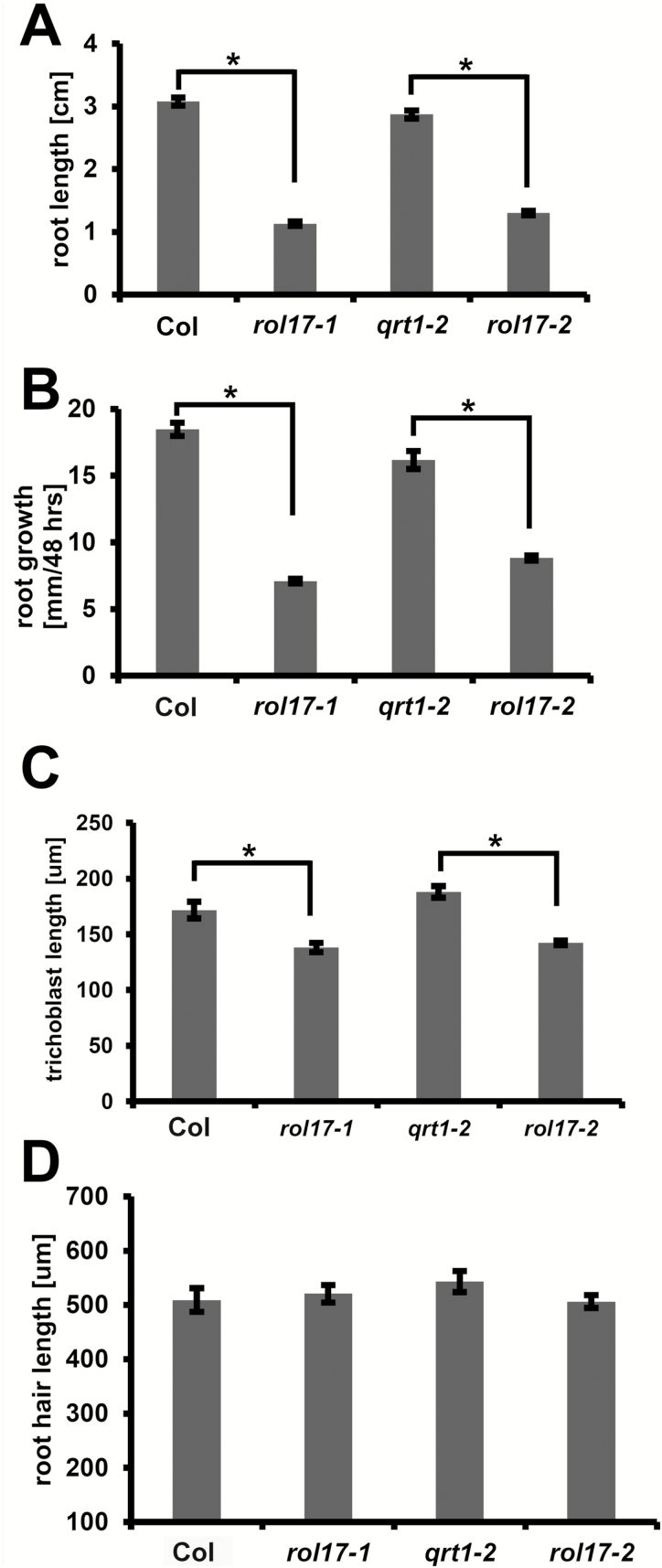
The two *rol17* alleles show similar root growth phenotypes. Seedlings were grown for 7 days on MS plates in vertical orientation. *qrt1-2* was used as a control because the SAIL T-DNA insertion mutant *rol17-2* contains the *qrt1-2* mutation in the background. The wild type (Col) was also used as a control. Root length (A), root growth (B), epidermal cell (trichoblast) length (C), and root hair length (D) were measured. Error bars represent SEM. Asterisks indicate significant differences (*P*<0.01; *t*-test, *n*>20).

Next, we aimed to identify the *rol17* locus. To this end, in the segregating F2 population of a second backcross of *lrx1 rol17* with *lrx1*, eight plants showing a *rol17* phenotype were selected, the plant material was pooled, genomic DNA was extracted, and whole-genome sequencing was performed. In parallel, the original *lrx1* mutant used for EMS mutagenesis (Diet *et al.*, 2006) was sequenced as well. A number of high-confidence polymorphisms in *lrx1 rol17* compared with *lrx1* were found in a region on the short arm of chromosome 1. Among these polymorphisms, four were found to change protein-coding sequences by missense mutations in the genes *wall associated kinase-like 4* (*WAKL4*), an *α/β-hydrolase* (*α/β-HYD*), *isopropyl malate synthase 1* (*IPMS1*), and *flavin-dependent monooxygenase 1* (*FMO1*) (Table 1), and were thus considered prime candidates for *rol17*. Seedlings of the segregating F2 population of the cross *lrx1 rol17* × *lrx1* were tested for recombination between the different candidate loci. In this way, the *wakl4* locus could be separated from the closely linked and thus co-segregating *α/β-hyd*, *ipms1*, and *fmo1* mutations, resulting in an *lrx1 wakl4* double mutant and an *lrx1 α/β-hyd ipms1 fmo1* quadruple mutant. The *lrx1 wakl4* double mutant showed a clear *lrx1* root hair phenotype and long roots, excluding *wakl4* as a candidate for *rol17* (see Supplementary Fig. S1). *lrx1 α/β-hyd ipms1 fmo1* quadruple mutant seedlings showed the *rol17* phenotype, with wild type-like root hairs and short primary roots; this line is subsequently referred to as *lrx1 rol17-1* (Table 2, Fig. 2A). For the three candidate genes *α/β-hyd*, *ipms1*, and *fmo1*, T-DNA alleles were obtained and analyzed (Table 1). Homozygous T-DNA mutants of *α/β-hyd* and *fmo1* revealed wild type-like root growth (Supplementary Fig. S2), confirming previous findings (Chen and Umeda, 2015). By contrast, the T-DNA mutant of *ipms1* developed shorter primary roots, similar to the *lrx1 rol17-1* mutant and, when crossed into the *lrx1* mutant background, caused suppression of *lrx1* (Fig. 2A, Fig. 3). This led us to conclude that *IPMS1* is allelic to *ROL17*. The *ipms1* T-DNA mutant is subsequently referred to as *rol17-2* (Table 2). The *rol17-1* polymorphism is in the fourth beta sheet in the N-terminal catalytic region (de Kraker and Gershenzon, 2011). This Pro186Leu codon change is at a residue that is conserved among eudicot plants, which overall show between 60% and 90% identity at the protein level. The T-DNA insertion in *rol17-2* is located in the 10th of 11 introns of the coding region (Supplementary Fig. S3) and has also been used by others (Field *et al.*, 2004).

**Table 1.** Candidate genes for the *ROL17* locus

**Gene identifier**	**Gene name**	**change in *rol17-1***	**T-DNA lines used**
At1G16150	*WAKL4*	Pro349Leu	SAIL_912_E08
At1G18460	*α/β-HYDROLASE*	Ser113Phe	SALK_024277C
At1G18500	*IPMS1*	Pro186Leu	SAIL_1175_E02
At1G19250	*FMO1*	Asp517Asn	SALK_026122C

Missense mutations in protein-coding genes identified by whole-genome sequencing, co-segregating with the *rol17* mutant phenotype.

**Table 2.** Genetic composition of lines used for detailed analyses

**line name**	**mutations in line**
*lrx1 rol17*	*lrx1, wakl4, α/β-hyd, ipms1, fmo1*
*lrx1 rol17-1*	*lrx1, α/β-hyd, ipms1, fmo1*
*rol17-1*	*α/β-hyd, ipms1, fmo1*
*lrx1 rol17-2*	*lrx1, ipms1*
*rol17-2*	*ipms1*

The listed mutations are present in the different lines.

In a next step, total RNA was extracted from 7-day-old seedlings and semi-quantitative RT–PCR was performed to investigate transcript abundance in the two alleles (Fig. 2B). Using a primer pair that amplifies an *IPMS1* sequence 5′ of the T-DNA insertion, a band of comparable intensity was detected in the extracts of the wild type and *lrx1 rol17-1*, whereas *lrx1 rol17-2* resulted in a weaker band; this observation suggested that the incomplete *ipms1* mRNA in *lrx1 rol17-2* is largely degraded, confirming previous findings (Field *et al.*, 2004). To ascertain that the intron-localized T-DNA insertion in *lrx1 rol17-2* indeed prevents proper splicing of the mRNA, primers located in the exons flanking the T-DNA insertion site were used for RT–PCR. No PCR product was observed in *lrx1 rol17-2*, confirming the absence of properly spliced mRNA, whereas transcript abundance was not affected in *lrx1 rol17-1* (Fig. 2B). All samples were free from contaminating genomic DNA, which results in a larger band owing to the presence of intronic sequence, and primers for *ACTIN2* (used as an internal standard) produced a strong band, confirming that RNA extraction and reverse transcription were successful in all samples.

### Mutations in *ROL17* affect root growth and epidermal cell elongation and cause hyposensitivity to AZD-8055

To compare the two *rol17* alleles in the *LRX1* wild-type background, the *lrx1 rol17-1* mutant was crossed with Col. In the F2 generation, plants with a short-root *rol17* mutant phenotype homozygous for wild-type *LRX1* were selected. Again, separation of either *α/β-hyd* or *fmo1* from *rol17-1* was not achieved, and *rol17-1* thus contains also these two mutations (Table 2). For the subsequent analyses, the *qrt1-2* mutant was used as the control for *rol17-2* because this SAIL T-DNA insertion line was produced in the *qrt1-2* mutant background (Sessions *et al.*, 2002). The short-root phenotype of *rol17-1* and *rol17-2* mutant seedlings (Fig. 3A) was further investigated. To exclude the possibility that the short-root phenotype is caused by a delay in germination, the growth rate of the root was determined in the different lines. Wild-type and *rol17* mutant seedlings were germinated and grown for 3 days, and the progression of the root tip was followed in the following 48 h. As shown in Fig. 3B, seedlings of both *rol17* alleles showed a reduced growth rate, indicating that root elongation, and not a defect in germination, causes the short-root phenotype. Measurements of epidermal cell length revealed a reduction in cell elongation in the mutants compared with the wild type (Fig. 3C), which is consistent with the reduced root growth of the *rol17* mutant seedlings. Interestingly, this impaired cell growth was not observed in root hairs, which were of comparable length in all lines (Fig. 3D).

**Fig. 4.**
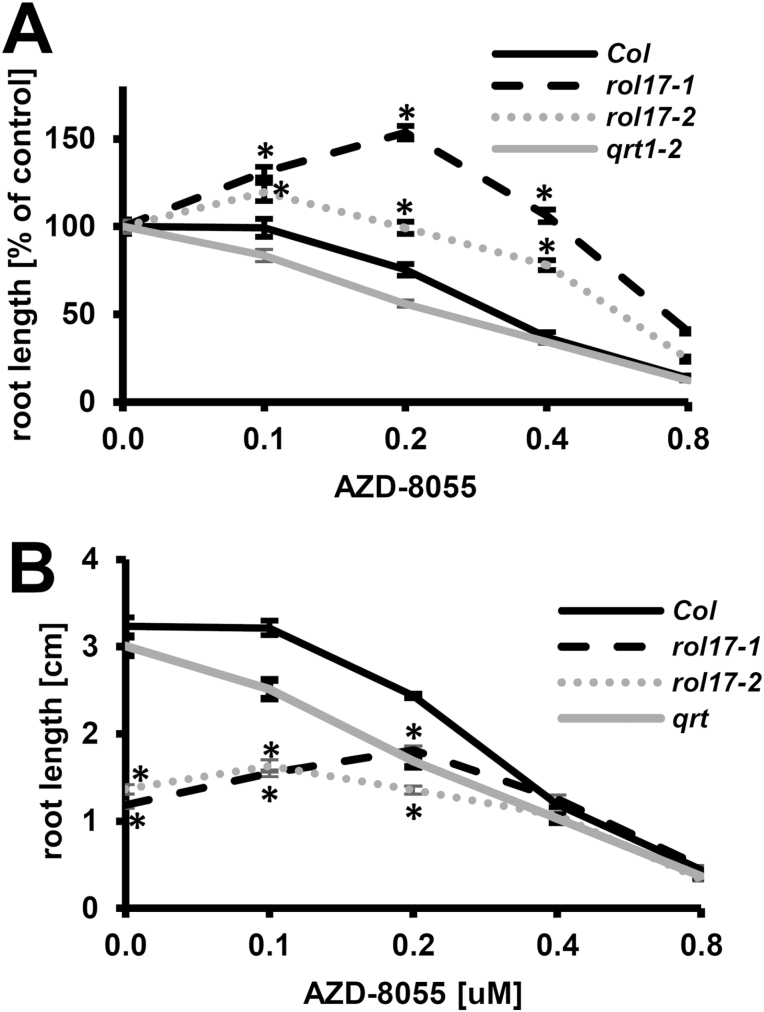
*rol17* mutations cause hyposensitivity to AZD-8055. Seedlings were grown for 8 days in vertical orientation on MS medium containing increasing concentrations of AZD-8055. (A) Change in root length, expressed as a percentage of root length in seedlings grown in medium lacking AZD-8055, and reduced sensitivity to AZD-8055 due to *rol17* mutations. (B) Absolute root lengths of the same seedlings analyzed in (A). At higher concentrations, all three lines have comparable root length. Asterisks indicate significant differences between the *rol17* mutants and their respective wild types (*P*<0.01; *t*-test, *n*>30). Error bars represent SEM.

AZD-8055 sensitivity was tested in the wild type and the two *rol17* alleles to confirm that mutations in this locus cause the hyposensitivity to the TOR inhibitor observed in the originally identified *lrx1 rol17* mutant. When seedlings were grown in the presence of increasing concentrations of AZD-8055, a weaker growth reduction was demonstrated in both *rol17-1* and *rol17-2* compared with their wild type (Col and *qrt1-2*, respectively) in the presence of the TOR inhibitor (Fig. 4A). At low concentrations of AZD-8055, both *rol17* alleles showed the absence of growth reduction and, rather, an increase in root length, which was particularly pronounced in *rol17-1*. In terms of absolute root length, the wild-type lines had longer roots than the *rol17* alleles only at lower AZD-8055 concentrations, and root lengths were comparable to those of the *rol17* alleles at 0.4 μM AZD-8055 or higher concentrations (Fig. 4B). This observation confirms that mutations in *rol17* cause altered sensitivity to the inhibition of the TOR kinase, indicative of a change in the TOR signaling network.

### Metabolomic alterations in *rol17* mutants

**Fig. 5.**
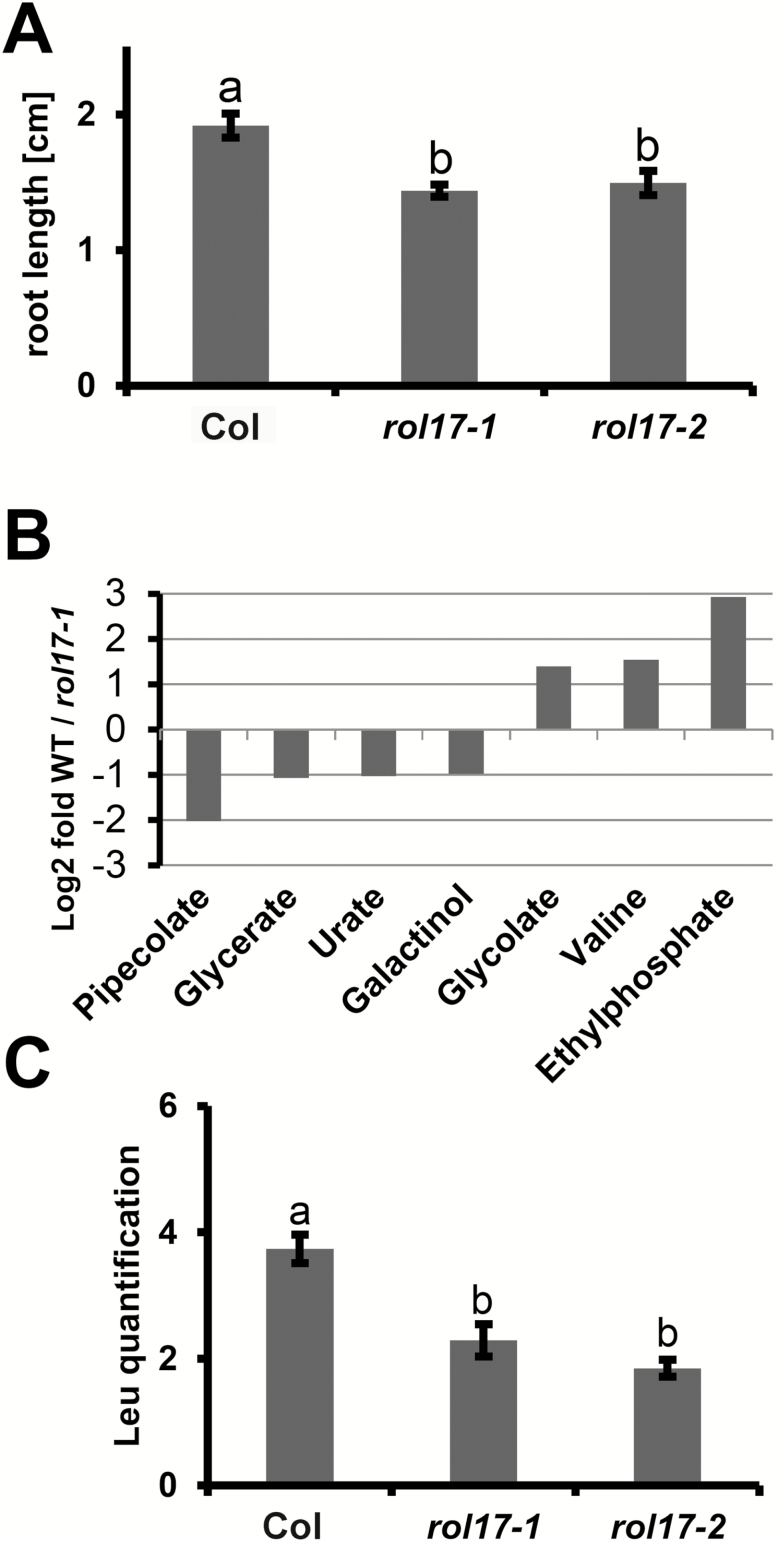
*rol17-1* alleles show changes in metabolite accumulation. (A) After being grown for 8 days in vertical orientation on HG medium, *rol17* mutants show reduced growth compared with the wild type (*P*<0.01; *t*-test, *n*>20). (B) Metabolites showing significant change in the *rol17-1* seedlings compared with the wild type. Criteria of more than 2-fold change and a significance threshold of *P*≤0.05 (*n*=5) were applied. Raw data on all metabolites tested can be found in Supplementary Table S1. (C) Total Leu content in seedlings grown for 8 days on MS medium. Leu was extracted from whole seedlings and quantification was done by LC-MS. The area under the peaks was used for quantification. Error bars represent SEM; different letters above the columns indicate significant differences (*P*≤0.001; *t*-test, *n*=4).

IPMS1 is involved in Leu biosynthesis, converting 2-oxoisovalerate to 2-isopropylmalate (de Kraker *et al.*, 2007). To test whether a mutation in *rol17* would change the accumulation of Leu and possibly other metabolites, a metabolomic analysis on 236 compounds (Clement *et al.*, 2018), including all amino acids, was performed on wild-type and *rol17-1* seedlings. For this purpose, plants were grown on HG medium, which is less rich in nutrients (Barberon *et al.*, 2008) than MS medium. The reduced root developmental phenotypes of both *rol17* alleles were also observed under these conditions (Fig. 5A). Only a few unambiguously identified metabolites showed significant divergence (≥2-fold change, *P*≤0.05) in accumulation between the two lines, among which valine (Val) was the only amino acid (Fig. 5B), comparable to previous findings (Field *et al.*, 2004; de Kraker *et al.*, 2007). Among the other metabolites identified, galactinol, glycerate, and pipecolate have previously been found to be influenced by the TOR network (Ren *et al.*, 2012; Caldana *et al.*, 2013). No significant difference in Leu content was observed between the wild type and *rol17-1* (1.14-fold reduction, *P*=0.06; Supplementary Table S1). There are differing reports on the effect of mutations in *IPMS1* on Leu accumulation, with no change or reduced Leu content compared to the wild type observed (Field *et al.*, 2004; de Kraker *et al.*, 2007).

**Fig. 6.**
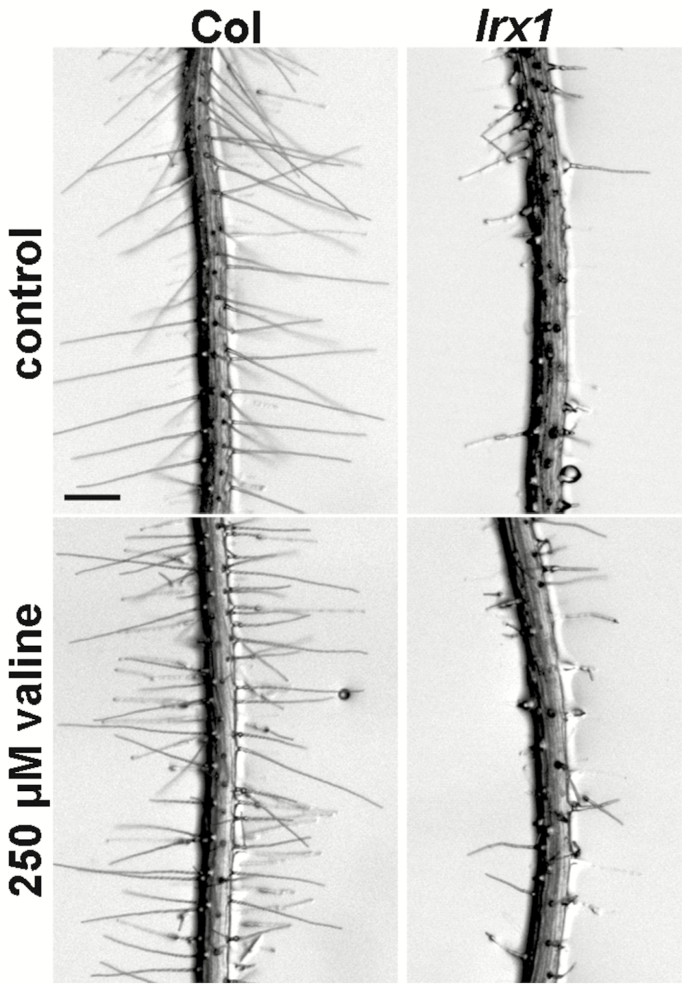
The *lrx1* mutant phenotype is not influenced by Val. Wild-type Col and *lrx1* mutant seedlings were grown in vertical orientation for 7 days on MS medium supplemented with 250 μM Val. The *lrx1* mutant phenotype developed unchanged under these conditions. Bar=0.5 mm.

As the plants used for these analyses were grown in different ways, we wanted to investigate whether nutrient availability might influence Leu accumulation. Wild-type, *rol17-1*, and *rol17-2* seedlings were grown in parallel on MS and HG media, and their Leu levels were determined. A difference in Leu content between the wild type and the *rol17* mutant seedlings could be detected only in seedlings grown on MS medium (Fig. 5C). Previous studies have shown that the exogenous application of Val interferes with plant growth (Wu *et al.*, 1994). To test whether the suppression of *lrx1* is related to the increased Val level observed in the *ipms1* mutants (in this study and Field *et al.*, 2004; de Kraker *et al.*, 2007), wild-type Col and *lrx1* mutant seedlings were grown on MS medium supplemented with increasing concentrations of Val. While Val induced the expected reduction in growth, the *lrx1* mutant phenotype was not altered at any Val concentration (Fig. 6).

## Discussion

Amino acid homeostasis is a crucial factor for coordinating growth processes with the nutrient status of the cell. Leu is a branched-chain amino acid (BCAA) that is produced from pyruvate in a series of enzymatic steps, most of which are shared with the synthesis of the BCAA Val (Galili *et al.*, 2016). Several enzymes encoded by the Arabidopsis genome have been identified to possess IPMS activity, including IPMS1 and IPMS2 (also referred to as MAML4 and MAML3, respectively) (Field *et al.*, 2004; de Kraker *et al.*, 2007). The two enzymes seem not to be completely redundant, because mutating *IPMS1* in *rol17* mutants causes conditional Leu deficiency. The wild type accumulates more Leu under nutrient-rich conditions, under which the general metabolism is likely to be higher and thus a sufficient supply of the amino acid pool is more critical.

In *rol17-1*, a Pro residue in the catalytic domain is changed to Leu (Supplementary Fig. S3). The strong conservation of this residue among plant IPMS proteins suggests that it is important for their enzymatic activity. This is supported by the very similar phenotypes seen with *rol17-2*, which lacks the C-terminal end of the protein and whose mRNA is reduced compared to the wild type. Previously, a third *ipms1* allele was analyzed with a T-DNA insertion in the seventh intron and no remaining mRNA detectable, representing a knockout allele (de Kraker *et al.*, 2007). Different groups have used different T-DNA insertion lines and the knockout allele does not have the strongest impact on the Leu pool (Field *et al.*, 2004; de Kraker *et al.*, 2007). This points to the possibility that growth conditions have a significant influence on Leu accumulation and possibly other metabolic activities.

### The *rol17* phenotypes are not induced by changes in Leu content

The most striking phenotype of both *rol17* alleles characterized here is a significant reduction in root growth (Fig. 3). This is at least partially explained by reduced cell expansion resulting in shorter cells, as exemplified by the decreased length of trichoblasts. Interestingly, root hair structures clearly do not show this reduced cell growth, indicating that the regulation of expansion of the root hair proper is distinct from the part integrated in the epidermis. The reduced root growth phenotype of the *rol17* mutants is observed independent of whether the Leu content is affected (as in MS-grown seedlings) or comparable in the wild type and *rol17* mutant lines (as in HG-grown seedlings), suggesting that the defect in plant growth cannot be simply explained by limited Leu availability. For the same reason, the function of Leu as a regulatory metabolite that mediates the regulation of gene expression (Hannah *et al.*, 2010) seems not to be of primary relevance here. Overexpression of the *IPMS1* homolog of Brassica in Arabidopsis does not induce increased amounts of Leu, possibly because of an autoregulatory feedback loop involving the C-terminal domain of the protein (Hagelstein and Schultz, 1993; Curien *et al.*, 2008; de Kraker and Gershenzon, 2011), but causes strong dwarfism in transgenic seedlings (Field *et al.*, 2006). This suggests that IPMS1-type proteins influence plant growth independent of their enzymatic activity.

### The TOR network is affected by the *rol17* mutations

Considering the importance of sensing nutrient availability and levels of metabolites, including amino acids, the TOR network is potentially a target mechanism by which IPMS1 might influence plant development. One well-studied activity is the control of translational activities (Leith and Hall, 2011; Laxman *et al.*, 2013; Schepetilnikov *et al.*, 2013). These are influenced by mRNA abundance but are also particularly dependent on amino acid availability. In animal cells, Leu and Glu influence TOR activity involving Rag-GTPases (Jewell *et al.*, 2015). The TOR network senses and influences amino acid homeostasis (Dobrenel *et al.*, 2016*a*) by modifying amino acid transport (Nicklin *et al.*, 2009) and protein turnover via autophagy (Liu and Bassham, 2010). Accordingly, altering TOR signaling by using RNAi constructs or treatment with the TOR inhibitor rapamycin also affects the abundance of amino acids including Leu (Moreau *et al.*, 2012; Ren *et al.*, 2012; Caldana *et al.*, 2013). In turn, it is conceivable that mutations affecting enzymes in amino acid metabolism have an effect on TOR signaling by altering translational activity. The hypothesis that mutations in *rol17* modulate plant development by influencing TOR signaling is supported by the observation that both *rol17* mutants characterized here show hyposensitivity to the TOR kinase inhibitor AZD-8055 (Figs 1B and 4). A change in sensitivity to TOR inhibitors has been used successfully as a selection criterion in genetic screens to identify components of the TOR network (Chan *et al.*, 2000; Li *et al.*, 2015), and our work provides evidence of such a role for IPMS1. This is further substantiated by the metabolomic analysis, which revealed few changes in *rol17-1* compared with the wild type. Among the seven compounds found to have significantly altered abundance between the wild type and *rol17-1* (Fig. 5B), galactinol, glycerate, and pipecolate have previously been identified as being regulated by the TOR network and show altered accumulation upon the inhibition of TOR activity (Moreau *et al.*, 2012; Ren *et al.*, 2012; Caldana *et al.*, 2013). The metabolic significance of the different metabolites is not always clear. For Val, the increase has also been observed with other *ipms1* mutants (Field *et al.*, 2004; de Kraker *et al.*, 2007) and can be explained by the redirection of the metabolic flux toward Val, which shares a large part of its biosynthetic pathway with Leu. Val and other BCAAs have a negative effect on root growth by inhibiting acetohydroxy acid synthase, the first common enzyme of the BCAA biosynthetic pathway (Wu *et al.*, 1994; Chen *et al.*, 2010). However, suppression of *lrx1* by *rol17* mutations is most likely not linked to the increased Val content, since the addition of Val to the growth medium did not suppress the *lrx1* root hair phenotype (Fig. 6). Galactinol is a precursor of raffinose-type oligosaccharides that are partly a means to transport sugar within the plant and have a major task as osmoprotectants (i.e. under cold stress; Peters and Keller, 2009; Egert *et al.*, 2013). Among other mechanisms, the TOR network has been shown to be important for osmotic stress responses (Deprost *et al.*, 2007), providing an explanation why it regulates the abundance of intermediary metabolites of these oligosaccharides.

Together with other amino acids, BCAA intermediates can be starting points for the biosynthesis of glucosinolates (GSLs), a group of sulfur- and nitrogen-containing secondary metabolites present in Brassicaceae, with over 40 different compounds in Arabidopsis (Field *et al.*, 2004). Upon wounding, GSLs can be metabolized by myrosinases, resulting in the accumulation of bioactive molecules with functions in defense against herbivores and pathogens (Bednarek *et al.*, 2009; Clay *et al.*, 2009; Kissen *et al.*, 2009; Bones *et al.*, 2015). GSLs and their degradation products also appear to be endogenous signals that influence plant development by modifying auxin perception, flowering time, the circadian clock, and by inhibiting plant growth (Kerwin *et al.*, 2011; Jensen *et al.*, 2015; Francisco *et al.*, 2016; Urbancsok *et al.*, 2017). A recent study revealed that the GSL 3-hydroxypropylglucosinolate influences plant development by interfering with the TOR network and has partly redundant activities with the TOR kinase inhibitor AZD-8055 (Malinovsky *et al.*, 2017). This presents a possible alternative way by which ROL17/IPMS1 might influence the TOR network. Our non-exhaustive metabolome analysis did not include GSLs, but a previous analysis did not reveal changes in GSLs in an *ipms1* mutant (Field *et al.*, 2004). In addition, several GSL degradation products (8MTO, 4MTB, nonanenitrile) were detected in our analysis and were not found to be altered by the *rol17-1* mutation. Hence, at this point, we do not have evidence that GSL metabolism would be changed by the *rol17* mutations and thus responsible for the observed alteration of the TOR network. However, a more detailed analysis of more metabolites in a larger sample size is required to draw a final conclusion on this point.

Suppression of the *lrx1* root hair defect by the *rol17* mutations (Fig. 2A) can be explained by the known effect of the TOR network on the expression of cell wall-related genes (Caldana *et al.*, 2013), cell wall architecture, and cell wall development (Leiber *et al.*, 2010). Since the TOR network is active in probably all cell types of an organism, the alteration in AZD-8055 sensitivity observed in the root growth of *rol17* mutants reflects a modified TOR network that is likely to affect root hair cells as well. For the coordination of the diverse mechanisms leading to cell growth, plants must regulate cell-wall-modulating activities, since cell wall expansion is a rate-limiting step in turgor-driven cell growth (Cosgrove, 2014). Previous work has shown that *lrx1* is suppressed by modifying TOR signaling (Leiber *et al.*, 2010), which has been confirmed in the present study with AZD-8055 suppressing the *lrx1* mutant phenotype (Fig. 1A). The TOR network can be significantly altered by proteins that are not directly implicated in the TOR signal transduction pathway but in a process that is under the influence of the TOR network. Several mutations blocking tRNA thiolation, which affects translational activity (Laxman *et al.*, 2013; Nedialkova and Leidel, 2015) and modifies the TOR network (Goehring *et al.*, 2003; Leidel *et al.*, 2009), cause suppression of *lrx1* (Leiber *et al.*, 2010; John *et al.*, 2014; Philipp *et al.*, 2014). The exact mechanism by which the TOR network influences the *lrx1* mutant phenotype remains to be demonstrated. LRX-type proteins have recently been identified as extracellular receptors of RALF peptides (Mecchia *et al.*, 2017) and seem to work in a process involving the FERONIA receptor kinase (Escobar-Restrepo *et al.*, 2007; Haruta *et al.*, 2014; Dünser *et al.*, 2018, Preprint). Downstream signaling of FERONIA involves the GTP-binding protein ROP2 (Duan *et al.*, 2010), which has recently been shown to influence TOR signaling and to physically interact with the TOR kinase (Schepetilnikov *et al.*, 2017). These findings lead to an emerging picture of a possible connection between LRX1, FERONIA, and the TOR network that will be the subject of future investigations.

## Supplementary data

Supplementary data are available at *JXB* online

**Fig. S1.** *wakl4* does not suppress *lrx1*.

**Fig. S2.** Root growth of T-DNA knockout lines of *rol17* candidate genes.

**Fig. S3.** IPMS1 protein comparison among different species.

**Table S1.** Raw data of metabolomic analysis.

**Table S2.** Primers used to identify the EMS polymorphisms.

**Table S3.** Primers used to identify the T-DNA mutations alleles.

**Table S4.** Primers used for RT–PCR.

Supplementary Figures S1-S3

Supplementary Table S1

## Abbreviations

BCAA: branched-chain amino acid
EMS: ethyl methanesulfonate
GSL: glucosinolate
HG: Hoagland
IPMS1: isopropylmalate synthase 1
Leu: leucine
LRX: leucine-rich repeat extensin
MS: Murashige and Skoog
rol: *repressor of lrx1*
TOR: Target of Rapamycin
Val: valine.
