# Supplementary material for: Mutations in the Arabidopsis ROL17/isopropylmalate synthase 1 locus alter amino acid content, modify the TOR network, and suppress the root hair cell development mutant lrx1
Source: J Exp Bot. 2019 Feb 8;70(8):2313–23. doi: 10.1093/jxb/ery463 (PMC6463047; doi:10.1093/jxb/ery463)
Supplement: Supplementary Figures S1-S3 [file ery463_suppl_supplementary_figures_s1-s3.pdf]

Supplementary data

A

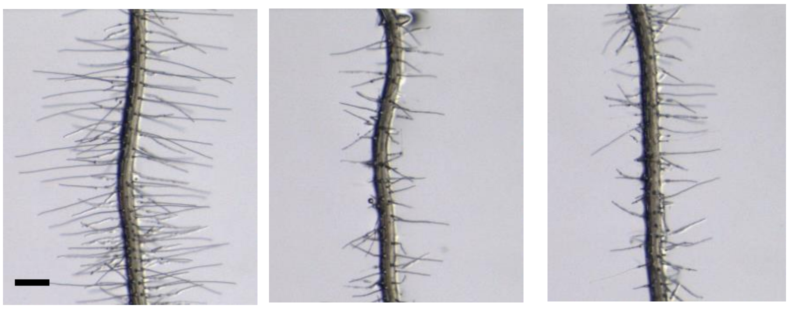

wild type *lrx1* *lrx1 wakl4*

B

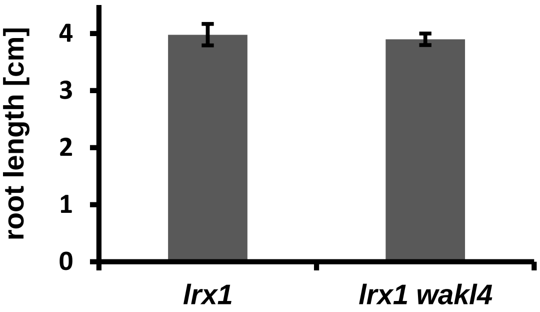

Fig. S1: *wakl4* does not suppress *lrx1*.
A) *lrx1* mutant seedlings develop aberrant root hairs compared to the wild type. This phenotype is not suppressed in the *lrx1 wakl4* double mutant line. Seedlings were grown for six days in a vertical orientation. B) *lrx1* and *lrx1 wakl4* seedlings grown for seven days on MS medium show similar root length. No signficiant difference was found (t-test, n>15, P>0.05). Error bars represent SEM. Bar= 0.5 mm.

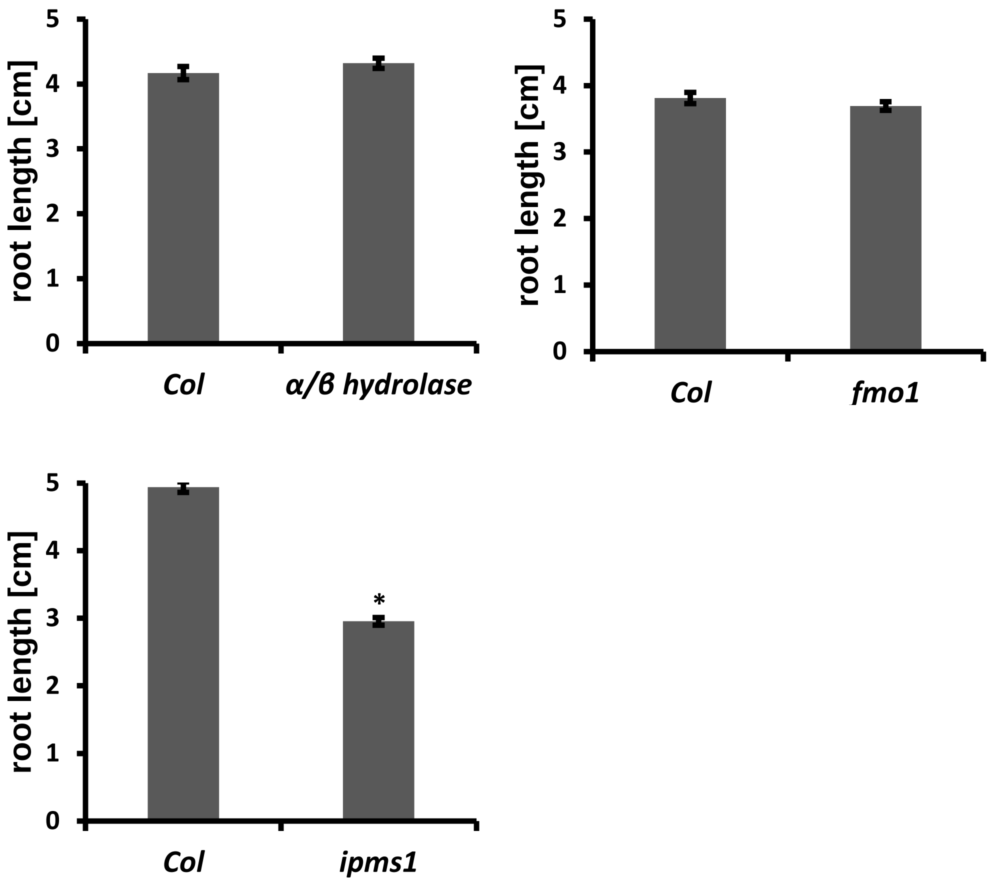

Fig. S2: Root growth of T-DNA knock-out lines of *rol17* candidate genes. Seedlings were grown for seven days in a vertical orientation. Only the *ipms1* T-DNA insertion line developed a short-root phenotype. Asterisks indicate significant difference (t-test, $n>20$, $P<0.01$). Error bars represent SEM.

Fig. S3

```
GmIPMS1   ------------------------------------------------------------   0
SlIPMS1   MASITANHP----------ISGKPLISFRPKNPLLQTQTLFNFKPSI----SKHSNSSF-   45
AtIPMS2   MESSILKSPNLSSPSFG--VPSIPALSSSSTSPF----SSLHLRSQNHRTISLTTAGKFR   54
AtIPMS1   MASSLLRNPNLYSSTTITTTSFLPTFSSKPTPIS----SSFRFQPSHHRSISLR-SQTLR   55
BiIPMS1   MESSILRNPMLSSPTTITT-PSLPSFSSKPSPL------SFRFPPSHHRS-SLR-IKSLR   51

GmIPMS1   MPTKTSTPSSQSPK-LSHLRPQYIPNHIPDSSYVRILDTTLRDGEQSPGATMTAKEKLDI   59
SlIPMS1   -------SIPV-VRCSIRRRPEYTPSHIPDPNYVRIFDTTLRDGEQSPGATMTTKEKLDV   97
AtIPMS2   VSYSLSASSPLPPHAPRR-RPNYIPNRISDPNYVRIFDTTLRDGEQSPGATLTSKEKLDI   113
AtIPMS1   LSCSISDPSPLPPHTPRRPRPEYIPNRISDPNYVRVFDTTLRDGEQSPGATLTSKEKLDI   115
BiIPMS1   LSCSLSDPSPP----LCRRRPEYIPNRISDPNYVRVFDTTLRDGEQSPGATLTSKEKLDI   107
                           : **:* *.:* * .***::**************:*:*****:

GmIPMS1   ARQLVKLGVDIIQPGFPSASNSDFMAVKMIAQEVGNAVDDDGYVPVIAGFCRCVEKDIST   119
SlIPMS1   ARQLAKLGVDIIEAGFPASSEADLEAVKLIAKEVGNGVYEEGYVPVICGLARCNKKDIDK   157
AtIPMS2   ARQLAKLGVDIIEAGFPAASKDDFEAVKTIAETVGNTVDENGYVPVICGLSRCNKKDIET   173
AtIPMS1   ARQLAKLGVDIIEAGFPAASKDDFEAVKTIAETVGNTVDENGYVPVICGLSRCNKKDIER   175
BiIPMS1   ARQLAKLGVDVIEAGFPAASKDDFEAVKTIAGTVGNAVDGDGYVPVICGLSRCNKRDIET   167
          ****.*****:*: ***::*: *: *** **  *** *  :******.*:.** ::** .

GmIPMS1   AWEAVKYAKRPRLCTSIATSPIHMEHKLRKSKDQVIQIARDMVKFARSLGCNDIQFGAED   179
SlIPMS1   AWEAVKYAKKPRIHTFIATSEIHMNYKLKMSRDQVVEKARSMVAYARSIGCEDVEFSPED   217
AtIPMS2   AWEAVKYAKRPRIHTFIATSDIHLKYKLKKSKEEVIEIARNMVRFARSLGCEDVEFSPED   233
AtIPMS1   AWDAVKYAKRPRIHTFIATSDIHLEYKLKKTKAEVIEIARSMVRFARSLGCEDVEFSPED   235
BiIPMS1   AWEAVKYAKRPRIHTFIATSDIHLEYKLKKSKDEVIEIARNMVKFARSLGCEDVEFSPED   227
          **:******:**: * **** **:::**: :: :*:: **.** :***:**:*::*. **

GmIPMS1   ATRSDREFLYEILGVVIEAGATTVNIADTVGIVMPLELGKLIVDIKDNTPGIANVIISTH   239
SlIPMS1   AGRSDPEFLYHILGEVIKAGATTLNIPDTVGYTVPEEFGQLIAKIKANTPGVEDVIISTH   277
AtIPMS2   AGRSEREYLYEILGEVIKAGATTLNIPDTVGITLPSEFGQLIADIKANTPGIQNVIISTH   293
AtIPMS1   AGRSEREYLYEILGEVIKAGATTLNIPDTVGITLPSEFGQLITDLKANTPGIENVVISTH   295
BiIPMS1   AGRSEREFLYQILGEVIKAGATTLNIPDTVGITLPSEFGQLIADIKANTPGIENVIISTH   287
          * **: *:**.*** **:*****:** **** .:* *:*:**..:* ****: :*:****

GmIPMS1   CHNDLGLATANTIEGARTGARQLEVTINGIGERAGNASLEEVVMALASKGDHALNGLYTR   299
SlIPMS1   CQNDLGLSTANTLAGACAGARQLEVTINGIGERAGNASLEEVVMALKCRGEQVLGGLYTG   337
AtIPMS2   CQNDLGLSTANTLSGAHSGARQVEVTINGIGERAGNASLEEVVMAIKCRGDHVLGGLFTG   353
AtIPMS1   CQNDLGLSTANTLSGAHAGARQMEVTINGIGERAGNASLEEVVMAIKCRGDHVLGGLFTG   355
BiIPMS1   CQNDLGLSTANTLSGAHSGARQVEVTINGIGERAGNASLEEVVMAIKCRGDHVLGGLYTG   347
          *:*****:****: ** :****:**********************: .:*::.*.**:*

GmIPMS1   INTRHILETSKMVEEYSGMHLQPHKPLVGANAFVHASGIHQDGMLKHKGTYETISPEEIG   359
SlIPMS1   INTQHILMSSKMVEEYSGLHVQPHKAIVGANAFAHESGIHQDGMLKHKDTYEIISPEDIG   397
AtIPMS2   IDTRHIVMTSKMVEEYTGMQTQPHKAIVGANAFAHESGIHQDGMLKHKGTYEIMSPEEIG   413
AtIPMS1   IDTRHIVMTSKMVEEYTGMQTQPHKAIVGANAFAHESGIHQDGMLKHKGTYEIICPEEIG   415
BiIPMS1   IDTRHIVMTSKMVEDYTGMQTQPHKAIVGANAFAHESGIHQDGMLKHKGTYEIICPEEIG   407
          *:*:**: :*****:*:*:: **** :******.* ************.*** :.**:**

GmIPMS1   HKRTTRIGIVLGKLSGSQALRKRLEELGYDLKEDEVDSVFWQFKAMAEKKKVVTDVDLKA   419
SlIPMS1   LNRANESGIVLGKLSGRHALQAKMLELGYEIEGKELDDLFWRFKSVAEKKKKITDDDLVA   457
AtIPMS2   LERSNDAGIVLGKLSGRHALKDRLNELGYVLDDGQLSNLFWRFKAVAEQKKRVTDADLIA   473
AtIPMS1   LERSNDAGIVLGKLSGRHALKDRLTELGYQLDDEQLSTIFWRFKTVAEQKKRVTDADIIA   475
BiIPMS1   LERSNDAGIVLGKLSGRHALKDRLTELGYVLDDEQLSSIFWRFKSVAERKKRVTDADIIA   467
           :*:.  ********* :**: :: **** :.  ::. :**:**::**:** :** *: *

GmIPMS1   LVSYKAFHAESIWKLGDLQVTCGTIGLSTATVKLVNIDGSTHVACSIGIGAVDSTYKAIN   479
SlIPMS1   LMSDEVFQPQFVWQLQNVQVTSGSIGLSTATVKLIDADGREHISCSVGTGPVDAAYKAVD   517
AtIPMS2   LVSDEVFQPEAVWKLLDMQITCGTIGLSTSTVKLADSDGKEHVACSVGTGPVDAAYKAVD   533
AtIPMS1   LVSDEVFQPEAVWKLLDIQITCGTIGLSTATVKLADADGKEHVACSIGTGPVDSAYKAVD   535
BiIPMS1   LVSDEVFQPEALWKLLDIQITCGTIGLSTATVKLADADGKEHVACSMGTGPVDSAYKAVD   527
          *:* :.*: : :*:* ::*:*.*::****:**** : **  *::**:* * **::***::

GmIPMS1   LIVKEPTKLLDYSLNSVTEGIGVNVTARVVICRENNHTSTYAFTEDANYPTFSGIAAEMD   539
SlIPMS1   LIVKVPVTLLEYSMNAVTQGIDAIASTRVLIRGENGHTSTHAVTGETIHRTFSGTGADMD   577
AtIPMS2   LIVKEPATLLEYSMNAVTEGIDAIATTRVLIRGDNNYSSTNAVTGESVERTFSGTGAGMD   593
AtIPMS1   LIVKEPATLLEYSMNAVTEGIDAIATTRVLIRGSNKYSSTNAITGEEVQRTFSGTGAGMD   595
BiIPMS1   LIVKEPATLLEYSMNAVTEGIDAIATTRVLIRGNNNYSTTNAITGEEVQRTFSGTGAGMD   587
          **** *..**:**:*:**:**.. .::**:*  .*  ::* *.* :    **** .* **

GmIPMS1   VVVSTVKAYLVALNKLLRWKESFRCA------------   565
SlIPMS1   IVISSVRAYVGALNKMMSFRKLMAKNNKPESSA-VV--   612
AtIPMS2   IVVSSVKAYVGALNKMLGFKEHTSTLSKTPLETNEVPA   631
AtIPMS1   IVVSSVKAYVGALNKMMDFKENSATK--IPSQKNRVAA   631
BiIPMS1   IVVSSVKAYVGALNKMLDFKENSTTK--IPSQNNKVPA   623
          :*:*:*:**: ****:: :::
```

Fig. S3: IPMS1 protein comparison among different species.
The IMPS proteins of *Arabidopsis thaliana* (At), *Glycine max* (Gm, soybean), and *Solanum lycopersicum* (Sl, tomato) are shown. Alignment was performed using the Clustal O(1.2.4) software. Identical positions are indicated in black. The asterisks, colons, and single dots refer to identical, conserved, and similar positions, respectively. The N-terminal catalytic domain is indicated by a red line on top, the regulatory domain important for the negative feed-back by Leu is indicated by a blue line. The Pro 186 changed to Leu in the *rol17-1* encoded protein is indicated by a red triangle, the T-DNA insertion in *rol17-2* in the intron between the codons for amino acids 338 and 339 by a blue triangle.

Supplementary Tables

Table S2: primers used to identify the EMS polymorphisms

Primer name	Primer sequence (5’→3’)	enzyme	dig. product [bp]
WAKL4_F	GCTCGTACGGCTACTTCTCTGG	Alu	WT: 394, 28<br>mut: 264, 130, 23
WAKL4_R	CAACAGTGCCGAACCTATAAGAACA		
α/β-HYD_F	GCTTGGACTATTCCTCTGGTGATG	BsmAI	WT: 261, 64<br>mut: 325
α/β-HYD_R	TCAGTAGTTCGGTTGGGCACGTCAT		
IPMS1_F	CTGTTGATGAGAATGGTTATGTTCC	AvrII	WT: 280, 104<br>mut: 384
IPMS1_R	CACCAAGAATCTCGTATAAGTACTC		
FMO1_F	TTTTTCTTCTTGACCGAGTCGGGAAT	ApoI	WT: 231, 12<br>mut: 206, 25, 12
FMO1_R	GTGGATGAGAAATTCAGATTACCAAG		

Primer pairs were used as indicated. The fmo1 polymorphism did not result in a change in a restriction site, making a point mutation in FMO1_F necessary (underlined) to introduce the ApoI polymorphism between FMO1 and fmo1.

Table S3: primers used to identify the T-DNA mutations alleles

Primer name	Primer sequence (5’→3’)	T-DNA specific primer
WAKL4_right	TACAGAGCCAGAAGAGTTTC	
WAKL4_left	GACTGGAACTTCCGTCTCAAG	Sail_Lb
α/β-HYD_ right	TTCAGACTGCCATTCTAGGAG	
α/β-HYD_ left	GGTTGATCCTCGTTTACTACC	Salk_Lb
IPMS1_ right	GCTAAGTACCATTTTCTGGCG	
IPMS1_ left	GACGCTTGACACCACAATATC	Sail_Lb
FMO1_ right	GAAGAAGGGATTGTGTTTGAAG	
FMO1_ left	GGACTAAAAGCTTCAAGGAGG	Sail_Lb
SALK_LB	GCGTGGACCGCTTGCTGCAACT	
SAIL_LB	CAGAAATGGATAAATAGCCTTGCTTC	

The primer pair for each gene amplified the wild-type copy. For amplification of the T-DNA insertion, Sail_Lb or Salk_LB primers were used together with the gene-specific primer as indicated, e.g. WALKF4_left with Sail_LB.

Table S4: primers used for RT-PCR

Primer name	Primer sequence (5’→3’)
IPMS1_RT_F1	AACTTGCTGACGCTGATGG
IPMS1_RT_R1	AAAAGAACCTAACTTCTGTCTGAC
IPMS1_RT_F2	GAAGTGACGATCAATGGAATTG
IPMS1_RT_R2	GCTCCTACAATAGCCTTATGAG
Act2_RT_F	AATGAGCTTCGTATTGCTCC
Act2_RT_R	GCACAGTGTGAGACACACC
